# Effect of dietary patterns differing in carbohydrate and fat content on blood lipid and glucose profiles based on weight-loss success of breast-cancer survivors

**DOI:** 10.1186/bcr3082

**Published:** 2012-01-06

**Authors:** Henry J Thompson, Scot M Sedlacek, Devchand Paul, Pamela Wolfe, John N McGinley, Mary C Playdon, Elizabeth A Daeninck, Sara N Bartels, Mark R Wisthoff

**Affiliations:** 1 Cancer Prevention Laboratory, Colorado State University, Fort Collins, CO 80523, USA; 2 Rocky Mountain Cancer Centers, Denver, CO 80218, USA; 3 Colorado Biostatistics Consortium, University of Colorado, Denver, CO, USA

## Abstract

**Introduction:**

Healthy body weight is an important factor for prevention of breast cancer recurrence. Yet, weight loss and weight gain are not currently included in clinical-practice guidelines for posttreatment of breast cancer. The work reported addresses one of the questions that must be considered in recommending weight loss to patients: does it matter what diet plan is used, a question of particular importance because breast cancer treatment can increase risk for cardiovascular disease.

**Methods:**

Women who completed treatment for breast cancer were enrolled in a nonrandomized, controlled study investigating effects of weight loss achieved by using two dietary patterns at the extremes of macronutrient composition, although both diet arms were equivalent in protein: high fat, low carbohydrate versus low fat, high carbohydrate. A nonintervention group served as the control arm; women were assigned to intervention arms based on dietary preferences. During the 6-month weight-loss program, which was menu and recipe defined, participants had monthly clinical visits at which anthropometric data were collected and fasting blood was obtained for safety monitoring for plasma lipid profiles and fasting glucose. Results from 142 participants are reported.

**Results:**

Adverse effects on fasting blood lipids or glucose were not observed in either dietary arm. A decrease in fasting glucose was observed with progressive weight loss and was greater in participants who lost more weight, but the effect was not statistically significant, even though it was observed across both diet groups (*P *= 0.21). Beneficial effects of weight loss on cholesterol (4.7%; *P = *0.001), triglycerides (21.8%; *P *= 0.01), and low-density lipoprotein (LDL) cholesterol (5.8%; *P *= 0.06) were observed in both groups. For cholesterol (*P *= 0.07) and LDL cholesterol (*P *= 0.13), greater reduction trends were seen on the low-fat diet pattern; whereas, for triglycerides (*P *= 0.01) and high-density lipoprotein (HDL) cholesterol (*P *= 0.08), a decrease or increase, respectively, was greater on the low-carbohydrate diet pattern.

**Conclusions:**

Because an individual's dietary preferences can affect dietary adherence and weight-loss success, the lack of evidence of a negative effect of dietary pattern on biomarkers associated with cardiovascular risk is an important consideration in the development of breast cancer practice guidelines for physicians who recommend that their patients lose weight. Whether dietary pattern affects biomarkers that predict long-term survival is a primary question in this ongoing clinical trial.

## Introduction

A lifestyle change can halve the risk of breast cancer recurrence and reduce the risk of breast cancer-associated mortality by one third. However, many clinicians do not recommend this strategy to their patients. The simple course of action, which generally is not discussed, is promoting a healthy weight, and for many individuals, this means weight loss. In this article, we begin to address this dormant opportunity in the clinical management of breast cancer with the goal of stimulating interest in generating the scientific evidence base required for considering weight management in clinical practice guidelines for the long-term survival of breast cancer patients.

A number of reports indicate that the prognosis for long-term survival after treatment for breast cancer is better in women who have a body weight for height, assessed by body mass index (BMI, body weight (kg)/(height (m))^2^), that is considered to be in the normal range (BMI, 18.5 to 24.9) versus women who are overweight (BMI, 25.0 to 29.9) or obese (BMI, ≥ 30.0) [1-11]. Consistent with those reports is the observation that weight gain after diagnosis increases risk for breast cancer recurrence, whereas weight loss in breast cancer survivors improves the chances of long-term survival [12,13]. If one takes the available epidemiologic and clinical data at face value, it prompts the question, why is relatively little attention paid to weight control in the clinical management of breast cancer after treatment.

Overweight and obesity are common problems in the United States, and little evidence indicates that prevalence is less in breast cancer survivors than in the population at large, which is estimated to be more than 60% [14-16]. Thus, given that the majority of breast-cancer survivors have excess weight as a risk factor, the population at risk is large. However, a number of challenges are faced by the physician. They include issues such as initiating a conversation about weight loss while recognizing the sensitivity of the subject and time constraints on office visits, which do not allow sufficient time to address the complexity of weight-management issues for each patient, including the knowledge and behavioral gaps related to diet and weight loss. Moreover, doctors may hesitate to emphasize weight loss, given the recognized 95% long-term failure rates of most weight-control efforts, making this information less a priority during the office visit (15-18). Additionally, because of lack of knowledge about the subject matter, basic questions such as "how should weight loss be achieved?" and "how much weight loss will provide benefit?" cannot be answered with confidence.

Although many studies have been reported about differences in effectiveness among various approaches to weight loss [17-25], relatively few studies have been conducted in a free-living population of breast-cancer survivors in the private-practice setting. The focus of this article is on whether diets that are the extremes of what most patients adopt for weight loss have any obvious deleterious effects in this population. Although treatment for breast cancer continues to improve, some first-line approaches still involve the use of agents with cardiotoxic potential [26-28]. Consequently, concern exists about cardiovascular risk factors and survival implications after breast-cancer treatment. This situation provided the rationale for the analysis of the blood-lipid profile, widely used for monitoring cardiovascular disease risk, and fasting glucose as an early indicator of insulin resistance, which were collected as a safety component of the investigation of the effects of diet composition and weight loss on biomarkers of long-term survival in breast-cancer patients after treatment.

For the analyses reported, magnitude of weight loss was dichotomized as being greater or less than the mean weight loss of the study population. This was done to address whether all participants are affected similarly or if the blood-biomarker outcomes are dependent on the magnitude of weight loss.

## Materials and methods

### Subjects

Women recruited for participation were from the same oncology practice and were at least 4 months after chemotherapy, radiation, and surgical treatment for breast cancer and considered clinically free of cancer. Accrual occurred from 2008 to 2010. Participants were referred by their medical oncologist and had a BMI in the overweight or obese class I range (BMI, 25 to 34.9 kg/m^2^).

### Eligibility

To be eligible, participants did not anticipate surgery over the study duration period; did not follow a special diet excluding foods or food groups; had not lost 4 or more pounds of body weight over the month preceding study initiation; did not take pharmaceuticals or supplements for weight management; were not being treated for diabetes or blood-glucose control; had no history of eating disorders; did not have digestive issues that might interfere with dietary intake, such as irritable bowel syndrome, Crohn disease, or diverticulitis; never had surgery involving constriction or removal of any portion of the gastrointestinal tract; had not been diagnosed with hepatitis B, C, or HIV; did not have implanted electronic devices such as a pacemaker; and did not use tobacco products. Participants also had to be willing to follow a dietary plan prescribed for the duration of the study; adhere to American Cancer Society alcohol guidelines (one or fewer standard drinks per day); maintain or increase physical activity as prescribed to achieve negative energy balance required for 0.5 to 1.0 kg weight loss per week; wear a pedometer and record daily activity; wear an accelerometer/heart-rate monitor for 2 weeks during the study; wear a body or swim suit and cap for body-composition testing; record food intake daily; attend up to 10 one-on-one clinic visits and five group visits over a 27-week period, and provide seven fasting blood samples and 3-day pooled urine samples.

### Study design

This study, referred to as CHOICE, compares the effects of opposing dietary patterns (carbohydrate and fat content at opposite extremes of popular weight-loss diet composition) on weight loss and body composition changes, as well as effects on biomarkers of metabolic and hormonal processes known to affect breast carcinogenesis and that are predictive of long-term survival. The details of the CHOICE research protocol have been published[29]. The data reported herein were collected as part of safety monitoring in a study designed as a nonrandomized, controlled trial. Assignment to treatment arm was based on dietary preferences because patient motivation is critical to successful weight loss, and dietary preferences can be strong determinants of adherence to a dietary plan. Participants were followed up for 6 months. During the course of the study, the withdrawal rate was 9.4% for the nonintervention control group, 12.9% for the low-carbohydrate group, and 11.3% for the low-fat group.

Demographic data and usual food intake via food-frequency questionnaire (VioFFQ; Viocare, Princeton, NJ) were collected at baseline. At the initiation of the study and at 4-week intervals thereafter, up to 6 months, anthropometry including weight, waist-to-hip ratio, BMI, body composition (BOD POD; Life Measurement, Inc., Concord, CA), and bioelectrical impedance (Tanita Corporation of America, Arlington Heights, IL) were obtained. Blood samples were obtained to assess cardiovascular-risk markers. These measures were assessed and compared at baseline and at 6 months in the control group. The clinical protocol was approved by the Institutional Committee for the Protection of Human Subjects. Written consent was obtained before enrolling participants.

### Dietary intervention

#### Control

Individuals not interested in joining the weight-loss arms of the study but who wished to participate were assigned to the nonintervention control group and were given the same information currently provided to all breast-cancer patients about the importance of avoiding posttreatment weight gain, and the health benefits of having a BMI in the normal range.

#### Intervention

Intervention participants follow a structured diet/physical activity program designed to create a weekly negative energy balance equivalent to 3, 500 kcal, after adjustments for metabolic adaptations that occur during extended periods of weight loss. The intervention groups received the same physical-activity protocol promoting the physical-activity guidelines and translated into step recommendations, but one of two diets that reflect commonly used weight-loss approaches that were identified in women attending a private oncology practice for long-term breast-cancer follow-up.

The diet plan for each group comprised a 28-day cycle of menus and recipes. The ingredients for each day's diet plan were entered into ProNutra Diet Analysis software. The macronutrient composition of the 28-day menu plan is shown in Table 1. The intended diet composition was derived from (a) identifying the most popular weight-loss programs undertaken by the clinic breast-cancer population; (b) conducting a systematic review of the literature to define the macronutrient composition of these diets and the actual intakes during weight-loss studies to determine feasible upper and lower limits; (c) defining an acceptable overlap that ensured diet separation (< 5% for fat and carbohydrate). The 28-day meal plans were designed for five calorie levels in each diet arm. The meal plans included interchangeable meal options (home-prepared recipes and meal instructions; eating out; and convenience meal options), educational material and a program incorporating weight-loss strategies based on a systematic review of those that support successful weight loss and maintenance and promote high levels of dietary adherence. The intervention was designed to reflect a feeding study in free-living individuals, where strict dietary structure is presented in a format that also offers enough flexibility to be adopted into daily living and by the families and social-support networks of participants.

**Table 1:** Dietary composition by diet group for a 28-day menu cycle (1, 200 kcal/day)

	Low carbohydrate, high fat	High carbohydrate, low fat
Calories	1, 204 ± 35	1, 186 ± 58
Carbohydrate (g)	100 ± 4 (32 ± 1)	193 ± 10 (62 ± 1)
Fat (g)	64 ± 3 (46 ± 1)	24 ± 2 (17 ± 1)
Protein (g)	68 ± 4 (22 ± 1)	62 ± 6 (20 ± 1)
Fiber (g)	17 ± 4	26 ± 5
Sodium (mg)	2, 113 ± 741	2, 586 ± 688
Cholesterol (mg)	244 ± 131	120 ± 85
Saturated fat (g)	11 ± 2	5 ± 2
Monounsaturated fat (g)	21 ± 3	5 ± 1
Polyunsaturated fat (g)	9 ± 2	5 ± 1
S/M/P ratio	1:2:1	1:1:1

Values are expressed as calories, grams, or milligrams ± standard deviation. Values in parentheses are percentage of energy ± standard deviation. Cycle menus were developed for the following calorie levels: 1, 200, 1, 400, 1, 600, and 1, 800 kcal/d.

### Laboratory measurements

Laboratory analyses were performed by Quest Diagnostics Inc. Fasting glucose was measured by using the hexokinase/glucose-6-phosphate dehydrogenase method with spectrophotometry [30]. Total cholesterol, HDL cholesterol, and triglyceride in plasma were determined enzymatically. For HDL, serum was combined with the 20% wt/vol polyethylene glycol in glycine buffer at pH 10.0 (25°C). All β-lipoproteins (LDL and VLDL) were precipitated. The HDL fraction (α-fraction) remained in the supernatant. The supernatant was then treated as a sample and assayed for cholesterol by an enzymatic method to determine HDL cholesterol value. Plasma LDL cholesterol was calculated by using following formula: LDL cholesterol = total cholesterol - (HDL cholesterol + (triglyceride/5)) [30-34].

### Statistical methods

Cohort characteristics at baseline were described as mean ± SD, and differences across diet groups were evaluated by using the global *F *test in a one-way analysis of variance. Maximum likelihood (ML) estimates of a repeated-measures model [35] by using complete cases were developed to assess the change over time in lipid measures; in other words, these were not intent-to-treat analyses. Separate slopes were estimated for diet group, and successful weight loss, defined as above or below the overall average weight loss in the two diet groups; that is, five slopes were present: control, high carbohydrate with weight loss greater than average, high carbohydrate with weight loss less than average, high fat with weight loss greater than average, and high fat with weight loss less than average. Linear contrasts were used to evaluate differences between selected slopes. Because visits were scheduled at roughly 1-month intervals, the slopes can be interpreted as the observed average change in a given measure for 1 month on treatment; the 6-month change can be estimated by multiplication. SAS version 9.2 (SAS Institute Inc., Cary, NC) was used for all statistical analyses. The Hochberg step-up procedure was use to adjust for multiple comparisons within each marker [36]. The algorithm is sort the *P *values from largest to smallest [37]: _*(k) *_, *P*_(*k*-1) _, ..., *P*_(1)_

$$\begin{array}{c} {\tilde{p}}_{\left(k\right)}=p_{\left(k\right)}\\ {\tilde{p}}_{\left(k-1\right)}=\text{min}\left({\tilde{p}}_{\left(k\right)},2p_{\left(k-1\right)}\right).\\ \vdots \\ {\tilde{p}}_{\left(1\right)}=\text{min}\left({\tilde{p}}_{\left(2\right)},kp_{\left(1\right)}\right). \end{array}$$

The adjustments are valid whether test statistics are independent or positively correlated.

## Results and Discussion

The effect of dietary pattern on blood-lipid and -glucose profiles was evaluated in 142 study participants. Data at baseline for the participants are shown in Table 2. No differences among groups were found in age, BMI, body weight, fat mass, disease stage, type of chemotherapy or hormonal therapy received, use of statins, or among the blood chemistries that served as end points in this investigation. To determine whether magnitude of weight loss had a moderating effect on the blood-lipid profile in the diet, the active treatment arms were subdivided into two groups corresponding to patients above or below the mean weight loss, which was 10 kg during the 6-month program. In brief, the range in weight loss over a 6-month period for the low-fat dietary group was 3.5 to 18.9 kg, and the range in body-fat loss was 3.4 to 19.3 kg. For the low-carbohydrate dietary group, the range in weight loss was 2.1 to 17.2 kg, and the range in body-fat loss was 1.2 to 18.5 kg. Based on self-reported pedometer counts, participants following the low-fat diet plan recorded 9, 661 ± 162 steps per day versus 8, 741 ± 170 steps per day for participants following the low-carbohydrate diet plan (mean ± SEM, *P *< 0.05).

**Table 2:** Baseline data profile by diet group

Variable	Control (*n *= 44)	Low fat (*n *= 50)	Low carb (*n *= 48)	*P* (Global *F *test)
Age (years)	56.61 ± 7.73	56.32 ± 8.08	55.19 ± 8.09	0.66
BMI	29.13 ± 2.92	28.37 ± 2.43	29.47 ± 2.65	0.12
Weight (lb)	175.53 ± 22.25	172.11 ± 18.54	176.74 ± 20.17	0.51
% Fat mass	43.37 ± 5.63	42.31 ± 4.91	43.65 ± 4.86	0.39
Glucose	83.60 ± 8.46	83.40 ± 9.22	86.33 ± 6.87	0.16
Cholesterol	204.72 ± 31.55	204.08 ± 42.41	201.66 ± 31.92	0.91
Triglycerides	133.28 ± 53.96	113.54 ± 58.72	139.13 ± 69.48	0.10
HDL	59.49 ± 16.10	66.30 ± 18.56	61.28 ± 15.87	0.13
LDL	118.53 ± 29.76	115.12 ± 37.56	112.57 ± 28.99	0.69
Chol/HDL ratio	3.63 ± 0.97	3.28 ± 1.05	3.50 ± 1.08	0.25
Years since diagnosis	5.43 ± 4.09	6.78 ± 6.21	8.40 ± 5.80	0.037
Stage 0 (%)	9.1	12.0	4.2	0.38
Stage I (%)	38.6	34.0	43.8	0.62
Stage IIA (%)	22.7	28.0	27.1	0.73
Stage IIB (%)	15.9	18.0	10.4	0.56
Stage IIIA (%)	9.1	4.0	6.3	0.61
Stage IIIB (%)	4.5	4.0	4.2	0.33
Stage IIIC (%)	0.0	0.0	2.1	0.41
Chemotherapy (%)	56.8	70.0	62.5	0.41
Hormonal therapy (%)	77.3	82.0	77.1	0.80
Statins (%)	15.9	18.0	20.8	0.83
Other cholesterol-lowering agents (%)	2.3	2.0	2.1	0.33

Carb, carbohydrate; HDL, high-density lipoprotein; LDL, low-density lipoprotein. Values are expressed as mean ± standard deviation.

**Figure 1:**
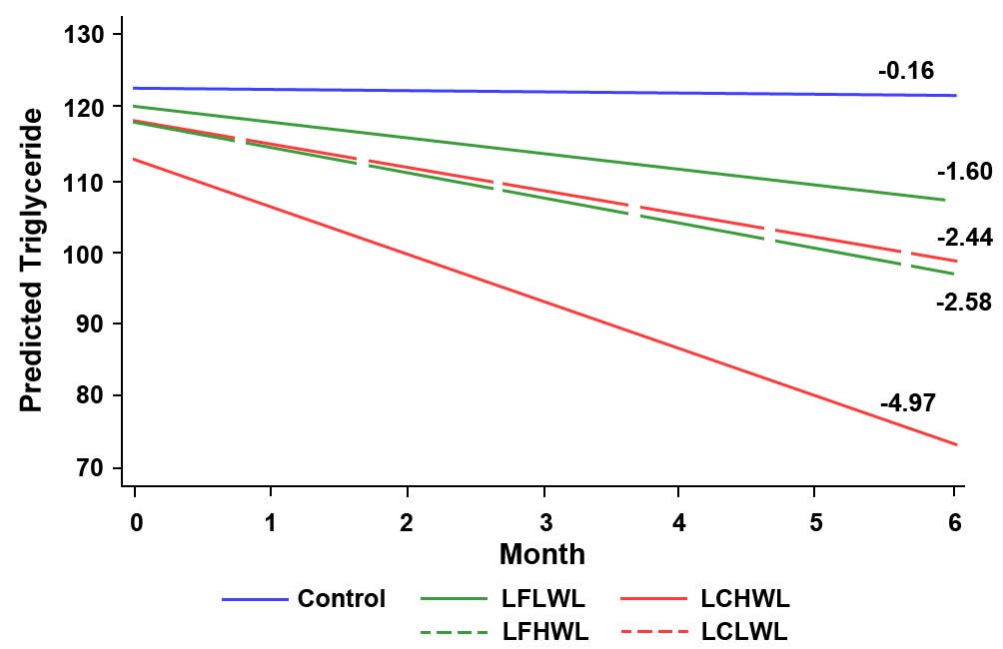
**Effect of weight loss and diet on plasma triglycerides**. Estimated slopes for predicted triglycerides (TGs) based on a repeated-measures model by group and whether weight loss was below or above the mean for the study population. Slope is the estimated monthly change in TGs. All slopes are significantly different from 0, with the exception of the control group. The Hochberg step-up procedure was used to adjust *P *values for multiple comparisons within each marker. Averaging over diet, the high- versus low-weight-loss slopes are different from each other (*P *= 0.01); with averaging over weight loss, the low-fat versus low-carbohydrate slopes are different from each other (*P *= 0.01), and within the low-carbohydrate diet, the high-weight-loss slope is significantly different from the low-weight-loss slope (*P *= 0.01). LFLWL, Low-fat low weight loss; LFHWL, low-fat high weight loss; LCLWL, low-carbohydrate low weight loss; LCHWL, low-carbohydrate high weight loss.

The research protocol involved monthly clinical visits during which fasting blood was drawn, and anthropometric data were collected. Blood was sent to the clinical laboratory routinely used by the office practice in which the weight management facility is located to base analysis and interpretation of results on the same source of data routinely used by the attending physicians. The detailed data from each clinic visit throughout the course of the study for each subgroup are shown in Table 3. Although inspection of those data is useful, it was decided that data interpretation would be facilitated by performing regression analyses of the entire set of data for each participant for each variable assessed. For ease of understanding, a representative regression is shown in Figure 1, with a detailed explanation of the data resulting from the regression analysis provided in the figure legend. The regression coefficients for all plasma analytes evaluated are summarized in Table 4, which also contains the statistical results.

**Table 3:** Mean levels of biomarkers over time by diet group

Month	0	1	2	3	4	5	6
**Glucose**							
Control	83 ± 1.3	-	-	-	-	-	84 ± 1.2
Low fat	83 ± 1.2	80 ± 1.1	82 ± 1.1	80 ± 1.0	79 ± 1.0	80 ± 1.1	80 ± 1.1
Low carb	86 ± 1.2	84 ± 1.1	83 ± 1.1	83 ± 1.0	82 ± 1.2	83 ± 1.1	82 ± 1.1
**Cholesterol**							
Control	205 ± 5.4	-	-	-	-	-	205 ± 5.4
Low fat	204 ± 5.1	178 ± 5.0	183 ± 4.9	187 ± 4.9	189 ± 4.9	189 ± 5.2	192 ± 5.2
Low carb	201 ± 5.2	184 ± 5.1	191 ± 5.0	193 ± 5.0	192 ± 5.0	194 ± 5.3	194 ± 5.3
**Triglyceride**							
Control	131 ± 9.3	-	-	-	-	-	126 ± 7.5
Low fat	114 ± 8.7	104 ± 6.9	105 ± 8.0	108 ± 7.0	99 ± 6.7	100 ± 7.0	100 ± 6.6
Low carb	138 ± 8.9	100 ± 7.0	112 ± 8.2	100 ± 7.2	100 ± 6.9	102 ± 7.1	97 ± 6.7
**HDL**							
Control	60 ± 2.6	-	-	-	-	-	60 ± 2.2
Low fat	66 ± 2.4	58 ± 2.0	60 ± 2.1	61 ± 2.3	64 ± 2.3	64 ± 2.2	66 ± 2.3
Low carb	61 ± 2.5	59 ± 2.0	60 ± 2.1	62 ± 2.4	63 ± 2.4	64 ± 2.3	65 ± 2.4
**LDL**							
Control	118 ± 4.9	-	-	-	-	-	118.93 ± 4.88
Low fat	115 ± 4.6	98 ± 4.5	103 ± 4.4	105 ± 4.3	106 ± 4.6	105 ± 4.8	106 ± 5.0
Low carb	112 ± 4.7	105 ± 4.6	108 ± 4.5	111 ± 4.4	108 ± 4.7	110 ± 4.8	108 ± 5.12
**CHOL/HDL**							
Control	3.62 ± 0.16	-	-	-	-	-	3.56 ± 0.13
Low fat	3.28 ± 0.15	3.19 ± 0.12	3.21 ± 0.12	3.26 ± 0.13	3.16 ± 0.13	3.10 ± 0.13	3.09 ± 0.13
Low carb	3.50 ± 0.15	3.24 ± 0.13	3.34 ± 0.13	3.26 ± 0.13	3.16 ± 0.13	3.17 ± 0.13	3.07 ± 0.13

Carb, carbohydrate; HDL, high-density lipoprotein; LDL, low-density lipoprotein.

**Table 4:** Estimated slope from repeated-measures models of biomarkers by time and diet group

Group weight loss	Glucose	CHOL	TRIG	HDL	LDL	CHOL/HDL
Control	0.03 ± 0.12	0.10 ± 0.47	-0.16 ± 0.62	0.20 ± 0.15	0.20 ± 0.40	0.00 ± 0.01
Low fat, low WL	-0.39^a^ ± 0.12	-1.56^a^ ± 0.56	-1.60 ± 0.59	0.41^a^ ± 0.17	-1.06 ± 0.54	-0.02 ± 0.01
Low fat, high WL	-0.60^a^ ± 0.14	-3.23^a^ ± 0.65	-2.58^a^ ± 0.68	0.00 ± 0.20	-2.30^a^ ± 0.63	-0.04^a^ ± 0.01
Low carb, low WL	-0.22 ± 0.14	0.01 ± 0.64	-2.44^a^ ± 0.66	0.69^a^ ± 0.19	0.15 ± 0.62	-0.03 ± 0.01
Low carb, high WL	-0.47^a^ ± 0.13	-2.65^a^ ± 0.59	-4.97^a^ ± 0.62	0.60^a^ ± 0.18	-1.42^a^ ± 0.57	-0.06^a^ ± 0.01
	
	***P^b^(adj^Pc^)***					
	
High vs. low WL averaged over diet	0.06 (0.21)	< 0.001 (0.001)	0.004 (0.01)	0.19 (0.38)	0.02 (0.06)	0.04 (0.16)
Low fat vs. low carb averaged over WL	0.21 (0.21)	0.07 (0.07)	0.007 (0.01)	0.02 (0.08)	0.07 (0.13)	0.43 (0.43)
High vs. low WL, low fat	0.21 (0.21)	0.04 (0.07)	0.24 (0.24)	0.12 (0.35)	0.13 (0.13)	0.21 (0.42)
High vs. low WL, low carb	0.14 (0.21)	0.002 (0.006)	0.003 (0.01)	0.760 (0.76)	0.06 (0.13)	0.10 (0.29)

Values are expressed as slope ± SEE. (*n *= 44 Control; *n *= 50 Low Fat; *n *= 48 Low Carb) ^a^Values within a column are statistically different from 0 at the 0.05 level. WL, weight loss; SEE, standard error of the estimate. ^b^The selected contrasts address the question of which slopes are different from each other. ^c^Adjusted *P *by using the Hochberg step-up procedure to control the family-wise type I error rate within each marker. Carb, carbohydrate; CHOL, cholesterol; HDL, high-density lipoprotein; LDL, low-density lipoprotein; TRIG, triglyceride.

Fasting blood glucose was assessed because elevations in this parameter can be an early indicator of developing insulin resistance, which is a risk factor for cardiovascular disease [38,39]; data on insulin, which are necessary for the computation of HOMA-IR, were not collected as part of safety monitoring, and therefore, further assessment is not possible at this time. The regression coefficients are negative for all weight-loss subgroups investigated. This means that fasting glucose decreased with progressive weight loss. The effect of weight loss was more pronounced than the effect of diet, although neither was statistically significant. The magnitude of the decrease in fasting glucose over time (slope of the line) was somewhat greater in participants who lost more weight when data were collapsed across diet groups: the difference in slopes was -0.46 ± 0.24 (*P *= 0.21). Similarly, the magnitude of the decrease in fasting glucose over time was somewhat greater in the low-fat arm than in the low-carbohydrate arm when data were collapsed across weight-loss groups: the difference in slopes was -0.31 ± 0.24 (*P *= 0.21). These findings are notable for several reasons. First, widespread debate is ongoing about the potential for high-fat diets to promote atherogenesis through the induction of insulin resistance, which can lead to elevated fasting glucose [25]. Second, relative to creating a microenvironment conducive to tumor growth, a repeated emergence of attention is noted in the metabolic re-programming that accompanies the development of cancer and recognition of the preference of many carcinomas for glucose or glutamine, which is actively taken up from the vascular system [40-44]. Hence, concern exists that diets rich in carbohydrates with a high glycemic load would stimulate tumor growth [45,46]. In the context of weight loss, no evidence was obtained to support either concern, as reflected by fasting glucose determined monthly over a period of 6 months; however, the differences between the slopes on diet averaged over weight were smaller (*P *= 0.06) than the differences between weight-loss groups averaged over diet (*P *= 0.21). This finding is consistent with the importance of weight loss to attain a body weight in the target range for height, which is generally stated as a body mass index (BMI, body weight in kilograms/height in square meters) between 18.5 and 24.9, although the target range can vary based on race [47].

Table 4 also shows the regression coefficients (estimated slopes) for plasma cholesterol, triglycerides, HDL cholesterol, and LDL cholesterol. These lipid and lipoprotein analytes are routinely used to monitor cardiovascular disease risk, but emerging evidence also indicates their potential relevance to tumor growth and progression [48]. A beneficial change in cholesterol, triglyceride, or LDL cholesterol is indicated by a negative regression coefficient, whereas higher levels of HDL cholesterol, indicated by a positive regression coefficient, are desirable for cardiovascular disease risk, although this may not be the case for cancer. The data in Table 4 show beneficial effects of weight loss on all four lipid analytes, as well as the ratio of cholesterol to HDL cholesterol. The degree of benefit was significantly greater for cholesterol (*P *= 0.001), triglycerides (*P *= 0.01), and LDL cholesterol (*P *= 0.06) in individuals who lost more than 10 kg versus those individuals who were less successful. A more-detailed inspection of the regression coefficients reveals that overall (irrespective of whether weight loss was below or above the mean), differential effects on the lipid analytes were found, depending on dietary assignment. For cholesterol (*P *= 0.07) and LDL cholesterol (*P *= 0.13), greater reductions appeared on the high-carbohydrate diet pattern; whereas, for triglycerides (*P *= 0.01) and HDL cholesterol (*P *= 0.08), changes in the beneficial direction were greater on the high-fat dietary pattern. Similar effects have been reported in a non-cancer survivor population [49].

Breast-cancer patients have elevated cardiovascular disease risk depending on their treatment; in patients who receive anthrocyclins, a well-known potential exists to induce cardiomyopathies with associated problems [28]. Because it has been reported that high dietary concentrations of lipid promote the metabolic processes that predispose to atherogenesis [25], safety monitoring focused on circulating lipids that are recognized indicators of cardiovascular disease risk is indicated. From the observed changes in lipid profiles, two findings are particularly noteworthy: (a) weight loss resulted in protective changes in the blood-lipid profiles, and (b) the beneficial changes occurred irrespective of dietary pattern. Because patient motivation is critical to successful weight loss, and dietary preferences can be strong determinants of adherence to a dietary plan, these findings indicate that from a safety perspective relative to cardiovascular disease risk, determined by the type of blood chemistries that attending physicians routinely have at their disposal, it is acceptable for patients to follow a dietary plan that meets their personal preferences during weight loss, provided that it is nutritionally balanced. Deeper inspection of the data shown in Table 4 indicates more-subtle differences of dietary pattern on specific lipid indicators; effects were associated with classification by whether weight loss was below or above the mean.

The findings on lipid metabolites also have implications related to mechanisms of tumor progression [50-54]. Although controversial, it has been reported in both epidemiologic studies and laboratory investigations that circulating levels of cholesterol, LDL-cholesterol, and HDL-cholesterol play a role in tumor development, tumor growth, and/or tumor aggressiveness, as summarized in [48]. In this regard, if causality is ultimately demonstrated, activities that limit availability of cholesterol and reduce the activity associated with cholesterol lipoprotein function would be considered beneficial. Hence, in view of the data shown in Table 4, our findings provide yet another mechanistic lead about the role of weight loss in promoting long-term survival after treatment for breast cancer.

### Limitations

Participants in this study were breast-cancer survivors from one clinical practice, which may limit the generalizability of the findings. We caution about the overinterpretation of these findings because the data were not the primary measures for the trial; we elected to control the type I error rate for the four comparisons done within each marker but not across markers. Another potential limitation of the study was that assignment to dietary arm was not randomized; however, this is likely to have translational value because individuals generally self-select dietary approaches that they prefer by which to lose weight.

## Conclusions

The work reported herein is a component of a systematic effort to increase awareness about the important contribution that weight loss and weight maintenance in the healthy range can offer to promote the long-term survival of breast-cancer patients. Given the prevalence of overweight and obesity, not only in the U.S. population as a whole, but also globally, and that a majority of women who have undergone treatment for breast cancer are overweight or obese, the importance of this issue is emphasized. The results of this investigation address a safety aspect of a question commonly asked of physicians by their patients: does it matter what dietary plan I choose to lose weight? Because an individual's dietary preferences can affect dietary adherence and weight-loss success, the lack of evidence of a negative effect of dietary pattern on cardiovascular risk is an important consideration in the development of clinical practice guidelines for physicians who recommend that their patients lose weight. Once weight is lost, questions similar to those being asked in this study must be addressed in the context of long-term weight maintenance.

## Abbreviations

BMI: Body mass index; HDL: high-density lipoprotein; HOMA-IR: homeostasis model assessment of insulin resistance; ML: maximal likelihood.

## Competing interests

The authors declare that they have no competing interests.

## Authors' contributions

HJT, SMS, PW, JNM, and MRW participated in the design and implementation of the study. DP, MCP, EAD, and SNB participated in the implementation of the study. All authors participated in the preparation of the manuscript.

## References

[B1] Ballard-Barbash R Anthropometry and breast cancer, body size: a moving target Cancer 1994 14 1090 100 10.1002/1097-0142(19940801)74:3+<1090::AID-CNCR2820741518>3.0.CO;2-X 8039144

[B2] Daling JR Malone KE Doody DR Johnson LG Gralow JR Porter PL Relation of body mass index to tumor markers and survival among young women with invasive ductal breast carcinoma Cancer 2001 14 720 9 10.1002/1097-0142(20010815)92:4<720::AID-CNCR1375>3.0.CO;2-T 11550140

[B3] Goodwin PJ Boyd NF Body size and breast cancer prognosis: a critical review of the evidence Breast Cancer Res Treat 1990 14 205 14 10.1007/BF01806329 2085672

[B4] Huang Z Willett WC Colditz GA Hunter DJ Manson JE Rosner B Speizer FE Hankinson SE Waist circumference, waist:hip ratio, and risk of breast cancer in the Nurses' Health Study Am J Epidemiol 1999 14 1316 24 10.1093/oxfordjournals.aje.a009963 10604774

[B5] La Vecchia C Negri E Franceschi S Talamini R Bruzzi P Palli D Decarli A Body mass index and post-menopausal breast cancer: an age-specific analysis Br J Cancer 1997 14 441 4 10.1038/bjc.1997.73 9020494 PMC2063359

[B6] McTiernan A Obesity and cancer: the risks, science, and potential management strategies Oncology (Williston Park) 2005 14 871 81 16053036

[B7] McTiernan A Irwin M Vongruenigen V Weight, physical activity, diet, and prognosis in breast and gynecologic cancers J Clin Oncol 2010 14 4074 80 10.1200/JCO.2010.27.9752 20644095 PMC2940425

[B8] Petrelli JM Calle EE Rodriguez C Thun MJ Body mass index, height, and postmenopausal breast cancer mortality in a prospective cohort of US women Cancer Causes Control 2002 14 325 32 10.1023/A:1015288615472 12074502

[B9] Radimer KL Ballard-Barbash R Miller JS Fay MP Schatzkin A Troiano R Kreger BE Splansky GL Weight change and the risk of late-onset breast cancer in the original Framingham cohort Nutr Cancer 2004 14 7 13 10.1207/s15327914nc4901_2 15456630

[B10] Reeves GK Pirie K Beral V Green J Spencer E Bull D Cancer incidence and mortality in relation to body mass index in the Million Women Study: cohort study BMJ 2007 14 1134 10.1136/bmj.39367.495995.AE 17986716 PMC2099519

[B11] Renehan AG Tyson M Egger M Heller RF Zwahlen M Body-mass index and incidence of cancer: a systematic review and meta-analysis of prospective observational studies Lancet 2008 14 569 78 10.1016/S0140-6736(08)60269-X 18280327

[B12] Harvie M Howell A Vierkant RA Kumar N Cerhan JR Kelemen LE Folsom AR Sellers TA Association of gain and loss of weight before and after menopause with risk of postmenopausal breast cancer in the Iowa Women's Health Study Cancer Epidemiol Biomarkers Prev 2005 14 656 61 10.1158/1055-9965.EPI-04-0001 15767346

[B13] Eliassen AH Colditz GA Rosner B Willett WC Hankinson SE Adult weight change and risk of postmenopausal breast cancer JAMA 2006 14 193 201 10.1001/jama.296.2.193 16835425

[B14] Flegal KM Carroll MD Ogden CL Curtin LR Prevalence and trends in obesity among US adults, 1999-2008 JAMA 2010 14 235 41 10.1001/jama.2009.2014 20071471

[B15] Rinaldi S Key TJ Peeters PH Lahmann PH Lukanova A Dossus L Biessy C Vineis P Sacerdote C Berrino F Panico S Tumino R Palli D Nagel G Linseisen J Boeing H Roddam A Bingham S Khaw KT Chloptios J Trichopoulou A Trichopoulos D Tehard B Clavel-Chapelon F Gonzalez CA Larrañaga N Barricarte A Quirós JR Chirlaque MD Martinez C Anthropometric measures, endogenous sex steroids and breast cancer risk in postmenopausal women: a study within the EPIC cohort Int J Cancer 2006 14 2832 9 10.1002/ijc.21730 16385576

[B16] Irwin ML Aiello EJ McTiernan A Bernstein L Gilliland FD Baumgartner RN Baumgartner KB Ballard-Barbash R Physical activity, body mass index, and mammographic density in postmenopausal breast cancer survivors J Clin Oncol 2007 14 1061 6 10.1200/JCO.2006.07.3965 17261853 PMC3839099

[B17] American Dietetic Association Position of the American Dietetic Association: weight management J Am Diet Assoc 1997 14 71 4 10.1016/S0002-8223(97)00024-2 8990423

[B18] Alhassan S Kim S Bersamin A King AC Gardner CD Dietary adherence and weight loss success among overweight women: results from the A TO Z weight loss study Int J Obes (Lond) 2008 14 985 91 10.1038/ijo.2008.8 18268511 PMC4005268

[B19] Sacks FM Bray GA Carey VJ Smith SR Ryan DH Anton SD McManus K Champagne CM Bishop LM Laranjo N Leboff MS Rood JC de Jonge L Greenway FL Loria CM Obarzanek E Williamson DA Comparison of weight-loss diets with different compositions of fat, protein, and carbohydrates N Engl J Med 2009 14 859 73 10.1056/NEJMoa0804748 19246357 PMC2763382

[B20] Bravata DM Sanders L Huang J Krumholz HM Olkin I Gardner CD Bravata DM Efficacy and safety of low-carbohydrate diets: a systematic review JAMA 2003 14 1837 50 10.1001/jama.289.14.1837 12684364

[B21] Greene LF Malpede CZ Henson CS Hubbert KA Heimburger DC Ard JD Weight maintenance 2 years after participation in a weight loss program promoting low-energy density foods Obesity (Silver Spring) 2006 14 1795 801 10.1038/oby.2006.207 17062810

[B22] Brehm BJ Seeley RJ Daniels SR D'Alessio DA A randomized trial comparing a very low carbohydrate diet and a calorie-restricted low fat diet on body weight and cardiovascular risk factors in healthy women J Clin Endocrinol Metab 2003 14 1617 23 10.1210/jc.2002-021480 12679447

[B23] Grieb P Kłapcińska B Smol E Pilis T Pilis W Sadowska-Krepa E Sobczak A Bartoszewicz Z Nauman J Stańczak K Langfort J Long-term consumption of a carbohydrate-restricted diet does not induce deleterious metabolic effects Nutr Res 2008 14 825 33 10.1016/j.nutres.2008.09.011 19083495

[B24] Adam-Perrot A Clifton P Brouns F Low-carbohydrate diets: nutritional and physiological aspects Obes Rev 2006 14 49 58 10.1111/j.1467-789X.2006.00222.x 16436102

[B25] Nordmann AJ Nordmann A Briel M Keller U Yancy WS Jr Brehm BJ Bucher HC Effects of low-carbohydrate vs low-fat diets on weight loss and cardiovascular risk factors: a meta-analysis of randomized controlled trials Arch Intern Med 2006 14 285 93 10.1001/archinte.166.3.285 16476868

[B26] Hooning MJ Botma A Aleman BM Baaijens MH Bartelink H Klijn JG Taylor CW van Leeuwen FE Long-term risk of cardiovascular disease in 10-year survivors of breast cancer J Natl Cancer Inst 2007 14 365 75 10.1093/jnci/djk064 17341728

[B27] Hooning MJ Aleman BM van Rosmalen AJ Kuenen MA Klijn JG van Leeuwen FE Cause-specific mortality in long-term survivors of breast cancer: a 25-year follow-up study Int J Radiat Oncol Biol Phys 2006 14 1081 91 10.1016/j.ijrobp.2005.10.022 16446057

[B28] Smith LA Cornelius VR Plummer CJ Levitt G Verrill M Canney P Jones A Cardiotoxicity of anthracycline agents for the treatment of cancer: systematic review and meta-analysis of randomised controlled trials BMC Cancer 2010 14 337 10.1186/1471-2407-10-337 20587042 PMC2907344

[B29] Sedlacek SM Playdon MC Wolfe P McGinley JN Wisthoff MR Daeninck EA Jiang W Zhu Z Thompson HJ Effect of a low fat versus a low carbohydrate weight loss dietary intervention on biomarkers of long term survival in breast cancer patients (CHOICE): Study Protocol BMC Cancer 2011 14 287 10.1186/1471-2407-11-287 21733177 PMC3150342

[B30] Fischbach FT Dunning MB III Manual of Laboratory and Diagnostic Tests 2009 Philadelphia: Lippincott Williams & Wilkins

[B31] Grundy SM Guidelines for cholesterol management: recommendations of the National Cholesterol Education Program's Adult Treatment Panel, II Heart Dis Stroke 1994 14 123 7 8044423

[B32] Grundy SM Cleeman JI Merz CN Brewer HB Jr Clark LT Hunninghake DB Pasternak RC Smith SC Jr Stone NJ Coordinating Committee of the National Cholesterol Education Program Implications of recent clinical trials for the National Cholesterol Education Program Adult Treatment Panel III guidelines Circulation 2004 14 227 39 10.1161/01.CIR.0000133317.49796.0E 15249516

[B33] Grundy S Third Report of the National Cholesterol Education Program (NCEP) Expert Panel on Detection, Evaluation, and Treatment of High Blood Cholesterol in Adults (Adult Treatment Panel III) 2002 Bethesda:National Institutes of Health 12485966

[B34] Balal M Paydas S Inal T Demir E Kurt C Sertdemir Y Validation of the Friedewald formula for the determination of low-density lipoprotein cholesterol in renal transplant recipients Ren Fail 2010 14 455 8 10.3109/08860221003658266 20446783

[B35] Verbeke G Molenberghs G Linear Mixed Models in Practice: An SAS Oriented Approach 1997 New York: Springer

[B36] Hochberg Y A sharper Bonferroni procedure for multiple tests of significance Biometrika 1988 14 800 10.1093/biomet/75.4.800

[B37] Westfall PH Tobias RD Rom D Wolfinger RD Hochberg Y Multiple Comparisons and Multiple Tests using the SAS System 1999 Cary, NC: SAS Institute Inc.

[B38] O'Malley G Santoro N Northrup V D'Adamo E Shaw M Eldrich S High normal fasting glucose level in obese youth: a marker for insulin resistance and beta cell dysregulation Diabetologia 2010 14 1199 209 10.1007/s00125-010-1693-0 20204321

[B39] Piche ME Lemieux S Perusse L Weisnagel SJ High normal 2-hour plasma glucose is associated with insulin sensitivity and secretion that may predispose to type 2 diabetes Diabetologia 2005 14 732 40 10.1007/s00125-005-1701-y 15765221

[B40] Shaw RJ Glucose metabolism and cancer Curr Opin Cell Biol 2006 14 598 608 10.1016/j.ceb.2006.10.005 17046224

[B41] DeBerardinis RJ Sayed N Ditsworth D Thompson CB Brick by brick: metabolism and tumor cell growth Curr Opin Genet Dev 2008 14 54 61 10.1016/j.gde.2008.02.003 18387799 PMC2476215

[B42] DeBerardinis RJ Lum JJ Hatzivassiliou G Thompson CB The biology of cancer: metabolic reprogramming fuels cell growth and proliferation Cell Metab 2008 14 11 20 10.1016/j.cmet.2007.10.002 18177721

[B43] Thompson CB Attacking cancer at its root Cell 2009 14 1051 4 10.1016/j.cell.2009.09.002 19766556

[B44] Wise DR Thompson CB Glutamine addiction: a new therapeutic target in cancer Trends Biochem Sci 2010 14 427 33 10.1016/j.tibs.2010.05.003 20570523 PMC2917518

[B45] Dong JY Qin LQ Dietary glycemic index, glycemic load, and risk of breast cancer: meta-analysis of prospective cohort studies Breast Cancer Res Treat 2011 14 287 94 10.1007/s10549-011-1343-3 21221764

[B46] Belle FN Kampman E McTiernan A Bernstein L Baumgartner KB Baumgartner R Dietary fiber, carbohydrates, glycemic index and glycemic load in relation to breast cancer prognosis in the HEAL Cohort Cancer Epidemiol Biomarkers Prev 2011 14 890 899 10.1158/1055-9965.EPI-10-1278 21430298 PMC3104475

[B47] WHO Expert Consultation Appropriate body-mass index for Asian populations and its implications for policy and intervention strategies Lancet 2010 14 157 63 10.1016/S0140-6736(03)15268-3 14726171

[B48] Llaverias G Danilo C Mercier I Daumer K Capozza F Williams TM Sotgia F Lisanti MP Frank PG Role of cholesterol in the development and progression of breast cancer Am J Pathol 2011 14 402 12 10.1016/j.ajpath.2010.11.005 21224077 PMC3069824

[B49] Foster GD Wyatt HR Hill JO Makris AP Rosenbaum DL Brill C Stein RI Mohammed BS Miller B Rader DJ Zemel B Wadden TA Tenhave T Newcomb CW Klein S Weight and metabolic outcomes after 2 years on a low-carbohydrate versus low-fat diet: a randomized trial Ann Intern Med 2010 14 147 57 10.7326/0003-4819-153-3-201008030-00005 20679559 PMC2949959

[B50] Cantafora A Blotta I Neutral lipids production, transport, utilization Anticancer Res 1996 14 1441 9 8694512

[B51] Tomiki Y Suda S Tanaka M Okuzawa A Matsuda M Ishibiki Y Sakamoto K Kamano T Tsurumaru M Watanabe Y Reduced low-density-lipoprotein cholesterol causing low serum cholesterol levels in gastrointestinal cancer: a case control study J Exp Clin Cancer Res 2004 14 233 40 15354407

[B52] Chang SJ Hou MF Tsai SM Wu SH Hou LA Ma H Shann TY Wu SH Tsai LY The association between lipid profiles and breast cancer among Taiwanese women Clin Chem Lab Med 2007 14 1219 23 10.1515/CCLM.2007.263 17663634

[B53] Kucharska-Newton AM Rosamond WD Mink PJ Alberg AJ Shahar E Folsom AR HDL-cholesterol and incidence of breast cancer in the ARIC cohort study Ann Epidemiol 2008 14 671 7 10.1016/j.annepidem.2008.06.006 18794007 PMC2566531

[B54] Kim Y Park SK Han W Kim DH Hong YC Ha EH Ahn SH Noh DY Kang D Yoo KY Serum high-density lipoprotein cholesterol and breast cancer risk by menopausal status, body mass index, and hormonal receptor in Korea Cancer Epidemiol Biomarkers Prev 2009 14 508 15 10.1158/1055-9965.EPI-08-0133 19190159

